# Just a ‘romantic idea’? A theory-based interview study on medication review implementation with pharmacy owners

**DOI:** 10.1007/s11096-022-01524-2

**Published:** 2023-01-13

**Authors:** Dorothee E. Michel, Antonella P. Tonna, Dorothee C. Dartsch, Anita E. Weidmann

**Affiliations:** 1 School of Pharmacy and Life Sciences, Robert Gordon University, Aberdeen, AB10 7GJ Scotland; 2 Cap Campus Pharmazie GmbH, Planckstraße 13, 22765 Hamburg, Germany; 3 Universität Innsbruck, Innrain 15, 6020 Innsbruck, Austria

**Keywords:** Community pharmacy, Framework for Implementation of Services in Pharmacy (FISpH), Implementation, Medication review, Pharmacy owners

## Abstract

**Background:**

Recent legal changes in Germany entitle patients on multiple medications to receive a medication review (MR). However, the provision of MRs is not mandatory and pharmacy owners decide whether to implement this service in their pharmacies.

**Aim:**

To determine pharmacy owners’ attitudes towards MRs, explore their experiences with MR implementation and examine their perceptions of barriers and facilitators towards implementation of MRs in community pharmacies.

**Method:**

Pharmacy owners were invited to participate in semi-structured interviews. Purposive sampling was used with selection criteria being MR-implementation stage, and geographical location of the pharmacy. The topic guide was based on a systematic review and the Framework for Implementation of Services in Pharmacy (FISpH). Interviews were recorded, transcribed verbatim and coded directly against the FISpH.

**Results:**

Twenty-one pharmacy owners were interviewed. Despite participants’ consistent positive attitude towards MRs, most believed that providing MRs on an economically viable basis would be challenging. Several practical suggestions emerged which would enable community pharmacies a smoother implementation of MRs. Suggestions included employing ‘change facilitators’, who visit and support implementing pharmacies; national awareness campaigns targeting patients and health professionals; reducing bureaucracy; continuing professional development; involving technicians in some MR-tasks; and offering an additional incentive to lower the initial implementation threshold.

**Conclusion:**

This research identified numerous factors that are likely to increase owners’ and managers’ support to the idea of MRs. This may be of interest to any country planning implementation of MRs.

**Supplementary Information:**

The online version contains supplementary material available at 10.1007/s11096-022-01524-2.

## Impact statements

- This theory-based study identified numerous practical suggestions that are likely to support MR-implementation in community pharmacies.
- Any country launching medication reviews in the community pharmacy setting might want to consider the suggestions to develop a successful implementation strategy.
- There is an urgent need to address owners’ concerns regarding the financial viability of the service in order to start the implementation process and thus make MRs available for more patients.

## Introduction

Growing evidence supports the benefit of pharmacists’ medication reviews (MRs) for patients in community settings [1–3]. The World Health Organization (WHO) recommends providing patients on multiple medications with an MR to reduce polypharmacy-related harm [2]. While MRs have been successfully implemented in several countries [4–6], their widespread implementation in Germany is lacking [7]. The German community pharmacy system is solely based on an item-related dispensing fee. Dispensing must follow a set of rules: Germany has 97 statutory health insurances (as of 2022) all of which have separate often differing contracts with generic drug manufacturers that community pharmacies must comply with. Dispensing of medical devices in turn is based on a range of separate contracts depending on the type of device and the insurance company agreement. In 2020, an amendment to the German legislation marked a paradigm shift towards pharmaceutical care as it entitled patients on multiple medications to receive an MR [8]. Parallel to this, a service-fund has been established guaranteeing a fixed remuneration by all health insurances for MRs, rather than solely item or device remuneration. However, MRs will not become mandatory for community pharmacies to provide, and pharmacy owners will need to decide individually whether to implement and offer the service in their pharmacies [9].

Implementation of complex interventions such as MRs is impacted by a multitude of factors that are context dependent and influence each other [10, 11]. A recent systematic review (SR) described stakeholders’ (e.g. pharmacists, prescribers, patients, and payers) experiences with MRs in community pharmacy. This SR identified publicity for the MR service, managerial support, patient feedback, pharmacists’ attitudes towards MRs and beliefs in MR outcomes, as key factors that influenced success or failure of the implementation [12]. Despite managerial support being one of the key factors, there is a paucity of literature reporting pharmacy managers’ and owners’ perspectives on the implementation of MRs.

This research aims to contribute to the limited evidence available and is underpinned by the Framework for Implementation of Services in Pharmacy (FISpH) [13], an implementation framework, which is based on the Consolidated Framework for Implementation Research [14], the Theoretical Domains Framework [15] and practice research in the community pharmacy setting. As any implementation effort strongly depends on the context in which it takes place, it is important to use a framework that is specifically suited for that context and FISpH fulfils these criteria as it comprises constructs identified in pharmacy practice research [16].

### Aim

This study aimed to determine pharmacy owners’ attitudes towards MRs, explore their experiences with MR implementation and examine their perceptions of barriers and facilitators towards implementation of MRs in community pharmacies.

### Ethics approval

All relevant ethical approvals for this study were obtained prior to commencing (Robert Gordon University, Scotland S290, 14.4.2021; Aerztekammer Hamburg, Germany 2021-300008-WF, 4.5.2021). Written informed consent was provided by participants prior to the study commencing.

## Method

### Study design

This study followed a qualitative phenomenological approach. Phenomenology sets out to explore participants’ views on, and experiences with, a given phenomenon (in this case: implementation of MRs) [17].

### Data collection tool development

A topic guide for semi-structured interviews was developed based on findings of this research team’s systematic review [12] and additional literature [18, 19]. All interview questions were mapped to the constructs of the Framework for Implementation of Services in pharmacy (FISpH) [13]. The topic guide [supplementary material 1] was piloted with two community pharmacy owners. Results from the pilot interviews were not included in the final data set. The topic guide included questions about participants’ knowledge and beliefs about MRs, whether they had any prior experience with MR-implementation or what would motivate them to consider implementation. Further questions covered the perceived demand for MRs, effectiveness of collaboration with doctors, and potential benefits of MRs.

### Sampling and recruitment

A background data questionnaire was designed to purposively sample community pharmacy owners across Germany. Potential participants were invited to the study through an announcement in a professional journal as well as by newsletters of pharmacy chambers. The questionnaire solely collected demographic data, e.g. federal state, town size, location of pharmacy (shopping centre, high street, small town etc.), pharmacy type, number of staff, and implementation stage. The main sampling criterion was the pharmacy’s current implementation stage. Stages follow the “Exploration Preparation Implementation Sustainment” (EPIS) Framework [11] which allows the classification of implementation stages. Further sampling criteria were location and size of the pharmacy. A member of the research team (DM) then contacted respondents by phone to arrange a convenient time for an online interview via Zoom® software (vs 5.6.6).

### Data collection

The principal researcher (DM) conducted all interviews after having received training in interview technique. The interviews were audio-recorded and transcribed *ad verbatim*. Transcripts were anonymised and then double checked for accuracy by a second researcher (AEW). Once no new themes relevant to this study’s objectives emerged, three more interviews were conducted to ensure data saturation was reached [20].

### Data analysis

The principal researcher (DM) used NVivo® 11 software to assist with data management. Data analysis followed the steps of a framework approach: *familiarisation; identifying a theoretical framework; indexing; charting; mapping and interpretation* [21]. Initially, the team *familiarised* themselves with the data by listening to recordings several times. Then one interview transcript was coded independently by 3 researchers (DM, DD and AEW) (*indexing*) against the pre-identified framework FISpH (*identifying framework*). This was discussed, taking care to consider the scope and interpretation of FISpH’s constructs within the context of German community pharmacies. The remaining transcripts were each analysed independently by 2 researchers. Discrepancies in coding were resolved by team discussion. The coded data was reorganised by construct (*charting*) and checked for consistency by two researchers. Finally, the principal researcher analysed the data in depth to identify implementation factors and associations between them (*mapping and interpretation*).

## Results

### Demographics

Sixty-seven pharmacists responded to the background data questionnaire. Respondents had classified themselves to be in the implementation stages *exploration* (n = 13), *preparation* (n = 9), *implementation* (n = 11), *sustainment* (n = 23) or had ticked *other* (n = 11) (Table 1). Twenty-one semi-structured interviews with pharmacy owners were conducted between 06-2021 and 09-2021. Two of the approached respondents were too busy to find time for an interview. The interviews lasted between 15 and 35 min.

**Table 1:** Characteristics of responding pharmacy owners, their pharmacies, and details of sampled participants

	Questionnaire responses	Participants sampled for interviewing
*Implementation stage*		
Exploration	13	4
Preparation	9	4
Implementation	11	4
Sustainment	23	7
Other	11	2
*Federal state*		
Baden-Württemberg	4	2
Bavaria	4	2
Berlin	1	1
Brandenburg	1	1
Bremen	0	0
Hamburg	3	1
Hesse	2	1
Mecklenburg-Vorpommern	3	2
Lower Saxony	5	4
North Rhine-Westphalia	15	2
Rhineland-Palatinate	9	1
Saarland	0	0
Saxony	9	1
Saxony-Anhalt	1	1
Schleswig–Holstein	6	1
Thuringia	4	1
*Town size*		
< 10,000	22	6
10,001–20,000	14	4
20,001–50,000	14	5
50,001–100,000	3	1
100,001–300,000	5	2
> 300,000	9	3
*Location*		
Shopping centre	3	0
Train station or airport	0	0
High street	10	2
Shopping arcade	9	4
Medical centre	14	5
Small town < 10,000	21	6
Other	10	4
*Pharmacy type*		
Single	36	11
1 + 1	16	5
1 + 2	11	4
1 + 3	4	1
*Number of pharmaceutical staff*		
< 5	22	7
5–9	38	11
10–14	3	2
15+	3	1

### Qualitative findings

**Fig. 1:**
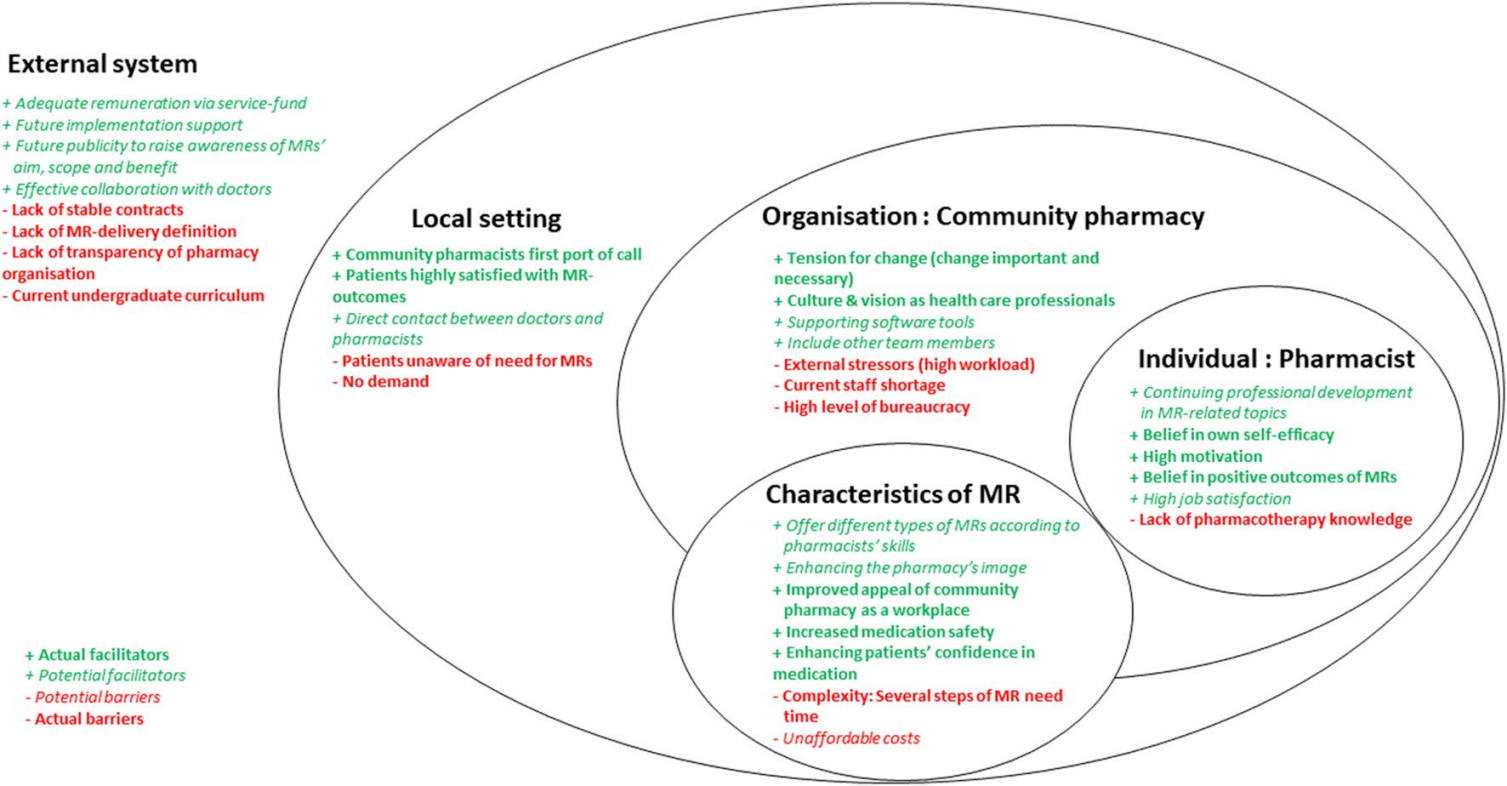
Implementation factors in the five domains of the Framework for Implementation of Services in Pharmacy: External system, local setting, organisation: community pharmacy, individual: pharmacist, characteristics of MR

All findings are presented within the domains of the FISpH. Implementation factors participants had experienced were either classified as *actual barriers* or *actual facilitators*. Factors which participants perceived would happen, suggested or planned to employ in the future were classified as *potential barriers* or *potential facilitators* (Fig. 1).

### External system

The external system concerns the wider political and healthcare system including regulating authorities and professional bodies. A great concern was a lack of stable and reliable contracts for community pharmacies.

“The last measures that have been in place, were announced, then withdrawn again. This means, something is commissioned but then in the end only half or a third is remunerated.” [P13, preparation]

Participants demanded a precise definition of the MR-service from the national pharmacy organisation together with adequate remuneration.

“[MR] is a romantic idea, but if the legal framework is vague and does not ensure the financial viability of the service, the entire thing does not stand a chance.” [P16, exploration]

The proposed future service-fund as well as the commissioning of MR-services as a result of changes to German legislation were deemed to be potential facilitators provided this would secure an adequate remuneration.

“I think [the service-fund] will come and we will get a foot in the door and then we can build on that.” [P6, sustainment]

However, the lack of transparency of the national pharmacy organisation (ABDA) regarding the nature of the negotiated pharmaceutical services with the insurers was perceived as an actual barrier.

“The national pharmacy organisation is lying very low, … and once [the contract] is finalised, we’ll all be taken by surprise because it will announce that MR delivery for all patients starts the day after tomorrow.” [P4, implementation]

Participants agreed that the professional bodies were responsible to offer implementation support and to raise awareness of MRs’ aims, scope and benefit.

“Maybe that's another suggestion to get the professional bodies to better pave the way and ... take everyone along step by step.” [P24, preparation]

“I believe that … it is important to use the media to demonstrate these benefits [of pharmacists’ MRs].” [P16, exploration]

The current undergraduate curriculum for pharmacy students emerged as an actual barrier as many graduating pharmacists lacked the pharmacotherapy knowledge to perform MRs.

“We [in German university education] are very focused on chemistry and the preparation of medicines and technology. … it’s not that they’re worthless, [that knowledge is] just not very useful for medication reviews.” [P19, sustainment]

Effective interprofessional collaboration with doctors was considered a potential facilitator as both the pharmaceutical and the medical perspective were necessary to optimise a patient's medication.

“Both doctor and pharmacist [are responsible… if you really look into [the medication] together, I think, the opportunities are tremendous.” [P12, sustainment]

### Local setting

The local setting includes patients, health care professionals and inhabitants of the community where the pharmacy is located. Many factors within the local setting were seen to be facilitators. All participants reported long standing and trustful relationships with their patients and the local community. Participants appreciated that patients regularly turned to them for advice as a first port of call.

“[Patients] don’t dare to ask the doctor, because they feel inferior … this inhibition threshold is far lower with us pharmacists.” [P12, sustainment]

Since the MR-service was largely unknown to the public, pharmacists regretted that patients did not feel a need for an MR and did not demand it.

“It never happened that a patient directly asked for [an MR]. I think the main problem is to notice the need to have their [patient’s] medication reviewed. … in addition [the patient] needs to know that an MR exists.” [P20, other]

However, once patients had been through a medication review, they were reportedly very satisfied with the outcomes.

“[Patients] thought it was great, thanked me a lot, gave a five-star rating on Google.” [P14, preparation]

The extent of the interprofessional collaboration between doctors and pharmacists varied. Still, many participants were optimistic that the collaboration would grow over time, and this would become a facilitator for MRs.

“The first couple years…were awkward. A doctor even said, ‘you’re throwing a spanner in my work’. However, after a personal dialogue, we came to a mutual understanding” [P17, sustainment]

### Organisation: community pharmacy

This domain captures all influences from within the pharmacy such as layout and workflow, staffing, teamwork, resources, organisational culture, and environmental stressors. Several participants described the current situation in the community pharmacy as not very conducive to the implementation of MRs since a heavy workload together with staff shortages rendered workdays very busy.

“It is impossible to allow a pharmacist to remove themselves to the office when we’ve got a shortage of pharmacists.” [P17, sustainment]

Participants had to cope with a high level of bureaucracy. Excessive documentation, complicated delivery contracts and frequent updating of the pharmacy’s licenses were recognized to be external stressors and consequently actual barriers.

“It’s definitely all the bureaucracy ... which I as the owner have to do outside of pharmacy hours.” [P18, implementation]

Nevertheless, many participants felt a strong tension for change towards services that made more appropriate use of their pharmaceutical knowledge.

“We can probably only survive if we do exactly that [MR]. As otherwise, Amazon will drop your asthma inhaler onto your balcony and then we’re done!” [P7, exploration]

The medication review service aligned well with several participants’ culture and vision for community pharmacy as they were hoping to enhance their standing as healthcare professionals.

"I hope, we're moving more towards a healthcare role. … not only doing logistics … I believe we can do more." [P20, other]

Many participants desired better software tools for MR delivery. The lack of supporting software made MRs time consuming and time was reported to be the scarcest resource overall.

“Well, you’d certainly need to rely on a certain amount of [software] support to make an MR feasible, implementable and to make it quicker.” [P6, sustainment]

Further support for the implementation of MRs could be generated by including other members of the pharmacy team. A participant suggested delegating some tasks to pharmacy technicians.

“Considering the staffing crisis, this can quickly turn the sophisticated pharmaceutical services we’re trying to establish into havoc within five years. That’s a real danger and can only be compensated for if we include the technicians somehow.” [P24, preparation]

### Individuals: pharmacists

This domain encompasses personal attributes of the pharmacists, their knowledge, values and motivation as well as reported self-efficacy and skills. Many participants thought an important barrier was the lack of sufficient pharmacotherapy knowledge.

“Clinical aspects, evidence base, and guidelines are not part of the education … if simple blood pressure targets aren’t known, and colleagues suggest calculating them as ‘100 plus age’ … what can you say?” [P6, sustainment]

Other participants argued that the necessary knowledge could be acquired and stressed the need for a continuing professional development.

“You have to keep at it. You really need to attend every [MR] seminar. Otherwise, you’ll forget.” [P22, implementation]

Many participants believed in their own self-efficacy but doubted their colleagues’ skills. A participant argued that pharmacists in general were sufficiently motivated and self-efficient to perform medication reviews well.

“I think that pharmacists have the skills to do it, that we have the heart to do it. It is not enough to have the data. You really have to want it.” [P15, other]

Potential positive outcomes for the patient safety by performing more medication reviews were strong motivators for many participants.

“If we consider the thousands of needless hospitalisations caused by adverse drug events, I can see a huge potential for cost savings for the health insurances.” [P17, sustainment]

In addition, MRs would contribute to high job satisfaction according to several participants.

“I chose this profession because I wanted to understand how [medicines] work and MRs represent the essence of the profession.” [P24, preparation]

### Characteristics of MR

Potential benefits of MRs for patients, community pharmacy and the healthcare system are covered in this domain. Other characteristics of MR-implementation and delivery that are covered here include complexity, adaptability, and costs.

Implementation of medication reviews was viewed as complex because the service consists of several steps that needed consideration and planning.

“You need to make an appointment … prepare everything, that’s half an hour, then you need to sit down with the patient for at least another half an hour and talk it through.” [P11, preparation]

The complexity of an MR entailed several associated costs that were not necessarily covered by the remuneration. Participants described this as a potential barrier.

“If an MR takes an hour, the pharmacist costs 50€ (50 USD), if you add a little for further operating costs, you will have to charge 70€ before you would start to break even.” [P19, sustainment]

To facilitate implementation, several participants suggested to start with simpler types of MR. This would make the MR more feasible for pharmacists with little prior experience.

“The most important aspect is … that you can say if the MR should be small, medium or large. … to allow you to come into contact with it more often … it lowers the inhibition threshold.” [P24, preparation]

Offering medication reviews would benefit community pharmacy as an organisation according to several participants. One participant was hopeful that the reputation of their pharmacy would improve.

“What are the benefits? To be honest, we certainly do hope for an image enhancement” [P6, sustainment]

Other participants thought MRs had increased the appeal of community pharmacy as a workplace.

“I think that [MRs] were an additional motivation to start working here … especially for young staff who don’t have a clear picture of the profession.” [P21, sustainment]

Medication reviews were believed to benefit patients as they increased medication safety for example by stopping unnecessary medicines.

“Sometimes medicines are being continued that should have been stopped. Why does [the patient] take this? It’s on the list.” [P12, sustainment]

Patients also benefitted from an increase in confidence, receiving information about their medication.

“Patients are inundated with information, and no one seeks the dialogue with them. … patients often get intimidated. In community pharmacy, we can solve problems, we can dispel fear.” [P15, other]

## Discussion

### Key findings

Overall, participating pharmacy owners had a positive attitude towards medication reviews regardless of their current implementation stage. In their opinion, the main benefits of an MR were an important contribution to patients’ medication safety and that performing MRs strengthened pharmacists’ role as health care professionals. In recognition that managerial support is a key factor to implementation, pharmacy owners suggested a variety of high-level practice structures that would enable them to implement MRs more readily. These include but are not limited to: the introduction of ‘change facilitators’, who visit and support pharmacies; a national awareness campaign; a reduction in the overly complicated dispensing rules, and the high level of bureaucracy in daily community practice; inclusion of pharmacy technicians in some MR-tasks; and the introduction of additional implementation incentives. Further well documented barriers such as remuneration and staffing concerns, required changes to the education of pharmacists and the need for growing collaboration with doctors have also been reported.

### Strengths and limitations

Use of FISpH to analyse the complex implementation considerations of pharmacy owners allowed for more meaningful results with the identification of potential solutions to perceived or actual barriers. Purposive sampling ensured that participants from all geographical regions and all implementation stages were included, which contributed to richness and diversity of the findings. Background description of participating owners and their pharmacies added to transferability of the findings. Coding and analysing independently by two researchers enhanced credibility, thus strengthening the study’s trustworthiness [22]. However, participants signing up for the study were likely to be more interested in and more open to MRs than others not signing up, thus possibly inducing a participation bias towards favourable views.

### External system

By the time of publication, many of the system-based barriers identified in this study had been addressed. A remuneration system for MRs in German community pharmacies had been agreed resulting in medication reviews now being available in community pharmacies whose owners opted to implement the service starting from June 2022 [23, 24]. It remains to be seen if this will help the MRs to transition from a mere ‘romantic idea’ to a nationally embedded pharmacy service. External implementation support to facilitate the process within individual community pharmacies was one other system-based factor requested by participants of this study. This can include ‘change facilitators’, who visit and support the implementing pharmacies, which has been a successful strategy in other countries [25–27]. The role of ‘change facilitators’ in those studies was to identify local barriers, motivate pharmacists, provide feedback, and to assist with individual implementation strategies, thus creating early wins [26]. Employing ‘change facilitators’ in Germany appears to be highly recommended.

### Local setting

Lack of awareness of the MR-service was perceived as major hindrance. Patients and professionals who are unaware of MRs will neither demand nor offer the service. Raising awareness on a wider national level will need a more collective strategy by German pharmacy organisations. This could include using mass media campaigns [28], which have been effective in increasing knowledge and awareness about other health topics as an international meta-analysis has shown [29]. On the local level, patients gave very positive feedback once they had received an MR, which was similarly reported in international studies [28, 30–33]. Capitalising on such patient-reports could be another way of spreading the word about the new MR-service.

Participants in our study took pride in being the first port of call for various health-related problems and believed that providing MRs will further strengthen this role. Despite the WHO supporting pharmacists’ expanded roles [2, 34], doctors remain sceptical [35], in particular some German doctors’ associations who are strongly opposing any pharmaceutical care [36]. However, international studies show that once a fruitful collaboration was established, doctors considered MRs valuable for their own work [18, 37].

### Organisation

German pharmacists’ shift towards a role as person-centred health care providers is not only important for public health but for community pharmacists themselves. This study’s participants perceived that their current business model, a remuneration system based on dispensing fees, was threatened due to the advent of online pharmacies in 2004. An increasing percentage of medicines is being purchased online [35] and is predicted to increase tenfold in the medium term [38]. This strongly contributed to the tension for change which pharmacy owners felt. The construct *tension for change* describes the *“degree to which the current situation is perceived as untenable or needing change”* [13, 39]. As long as all involved are comfortable in a given situation, no change is likely to occur [39, 40]. The desire for change was further increased by overly complicated dispensing rules, and bureaucracy in general. Both consumed valuable time which could be of better use for person-centred care. Internationally, there have been several calls to change pharmacy practice and to adapt community pharmacy to present-day health needs of the society, delivering more person-centred care [41, 42]. This includes restructuring the workflow and adjusting team members’ roles. Workflow changes and including the entire team have been identified as important facilitators towards MR-provision in a German study by Waltering et al. [43]. Our study’s participants suggested to include technicians in some of the MR-tasks, a strategy that had been used in the USA to facilitate scheduling, billing and documentation of MRs [44] and was shown to increase MR completion rates [45].

### Individual

Continuing professional development for pharmacists was deemed important by this study’s participants, particularly since the current German undergraduate curriculum does not include pharmacotherapy in any great depth [46]. A need for up-skilling qualified pharmacists in pharmacotherapy for providing MRs has been reported [47], even from countries such as the UK [48] or USA [44] where clinical pharmacy and pharmacology account for more than 45% of the undergraduate curriculum as compared to 12% in Germany [49, 50]. Participants thought that strengthening and using pharmacotherapy knowledge for MRs would contribute considerably to higher job satisfaction, a suggestion supported by findings of an Australian survey in which opportunities to apply knowledge was named as the most important criterion for an ideal job [51].

### Characteristics of MR

Pharmacy owners held positive views of MRs and believed in positive MR outcomes, which aligns with views of employed pharmacists reported in the literature [12, 28, 32]. Positive views can act as facilitator for implementation [15]. However, the complexity of the intervention was perceived as hindering and pharmacy owners were concerned that the recent remuneration model would not cover the costs of implementing and sustaining MR-delivery. It might be worthwhile to consider an additional implementation incentive (e.g. extra professional development credit points for the first 5 MRs performed) to lower the implementation threshold. With every medication review performed experience grows, and less time will be needed for an MR [52], which in turn will add to the financial viability of the service.

### Future research

This study has identified implementation factors across all domains which build a sound basis for a nationwide implementation strategy [53]. Future research should utilise stakeholders’ knowledge to seek consensus on the development of a practice strategy [54].

## Conclusion

Despite participants’ overall positive perceptions about medication reviews, some scepticism remained whether, and how implementation would be feasible. This study’s participants made several practical suggestions for structures that would enable them to implement MRs more readily and adapting community pharmacy to present-day health needs. Any country planning to develop a nationwide implementation strategy might want to consider these factors to address owners’/managers’ concerns and lower the initial implementation threshold.

## Supplementary Information

Below is the link to the electronic supplementary material.

Supplementary file1 (DOCX 40 kb)
