# Publisher Correction to: Just a ‘romantic idea’? A theory-based interview study on medication review implementation with pharmacy owners

**DOI:** 10.1007/s11096-023-01546-4

**Published:** 2023-03-15

**Authors:** Dorothee E. Michel, Antonella P. Tonna, Dorothee C. Dartsch, Anita E. Weidmann

**Affiliations:** 1 School of Pharmacy and Life Sciences, Robert Gordon University, Aberdeen, AB10 7GJ Scotland; 2 Cap Campus Pharmazie GmbH, Planckstraße 13, 22765 Hamburg, Germany; 3 Universität Innsbruck, Innrain 15, 6020 Innsbruck, Austria

**Correction to: International Journal of Clinical Pharmacy** https://doi.org/10.1007/s11096-022-01524-2

In the original publication of the article, the affiliation of the second author “Antonella P. Tonna” was incorrectly published.

The correct affiliation of the author “Antonella P. Tonna” is “School of Pharmacy and Life Sciences, Robert Gordon University, Aberdeen AB10 7GJ, Scotland”.

The original article has been corrected.

