# Supplementary material for: Just a ‘romantic idea’? A theory-based interview study on medication review implementation with pharmacy owners
Source: Int J Clin Pharm. 2023 Jan 13;45(2):451–60. doi: 10.1007/s11096-022-01524-2 (PMC10147800; doi:10.1007/s11096-022-01524-2)
Supplement: Supplementary file 1 — Supplementary file1 (DOCX 40 kb) [file 11096_2022_1524_MOESM1_ESM.docx]

**(Topic guide Pharmacy Owners): Date: Time: participant ID:__**

| **linked to objective** | **(main question)** | **(probing / explanation)** | **theoretical underpinning:  FISpH (Moullin 2016)** |
| --- | --- | --- | --- |
|  | Hello, my name is Dorothee Michel. This study is part of a research project at Robert Gordon University, Scotland.  First, I would like to thank you for participating in this study! | You can withdraw consent at any point of time without giving any reason.  As described in the consent form, this interview will be recorded.  Is this still okay with you? - Then I will start recording now. | **Welcome**  **start recording!** |
| 1 | Could you start telling me what you know about medication reviews (MRs)? | Explanation, only if the participant asks about it:  A medication review is the structured evaluation of a patient’s medicines, gathering further medicine related information in a patient interview (type 2a). Clinical data will be considered additionally in a medication review type 3. Subsequently, potential or manifest medicine related problems will be evaluated, prioritised and solutions suggested. Finally, interventions will be discussed and agreed with the patient (and doctor where necessary). | IV 2 knowledge about MRs |
| 1 | Do you think MRs are a necessary service? | Why (not)? Necessary for patients / pharmacists?  In your opinion, who is responsible for medication safety? | III 8a tension for change |
| 3 | Do you think pharmacists are well prepared for such a more clinical role? | Why (not)?  How does this impact on implementation of MRs in your pharmacy? | IV 5a+5c technical and interpersonal skills |
| 4 | Have you got any experience with implementation of MRs in your pharmacy? | **If yes:** What was helpful? What hindered the implementation?  Which type of MR have you conducted? (PCNE 1/ 2a,b/3)? probe: **How did you proceed?  If no:** Do you plan to offer MRs? Why (not)? What would motivate you to offer MRs?  If necessary, clarify:  **Type 1:** simple MR, using patient record in pharmacy; enables identification of interactions, some side effects, some adherence issues, unusual dosages  **Type2a**: intermediate MR with patient interview and patient record; in addition to type 1, identification of adherence issues, interactions with food, issues with effectiveness, problems with OTC medicines, untreated conditions, or medicines without indication. **Type 2b**: intermediate MR with patient record and clinical data; similar to 2a, but different focus, as there is no patient interview; effectivity would focus on clinical parameters. **Type 3**: comprehensive MR with patient interview, patient record and clinical data; all named above. | III 14 experience |
| 4+5 | Are there other tasks in your pharmacy MRs will compete with? | What are your priorities? Priorities in your team? | III 8c relative priority+ III 12 environmental stressors |
| 4+5 | Do you think there is a patient demand for MRs? | Why? OR Can you tell me more about it? | II local 6 patient needs / II local 5 demand |
| 4+5 | How do doctors react to your MRs?  OR *How will doctors react to you offering MRs?* | Are you connected with doctors in your area? What type of information do you exchange? | II local 2 interprofessional network & collaboration |
| 2+5 | Do you think offering MRs gives you an advantage over your competitors? | With hindsight to customer numbers? Professional reputation? Staff satisfaction? Did this influence your decision (not) to implement MRs? | II local 7 peer pressure / II local 3 community’s perception about pharmacy |
| 2 | Would you prefer to implement other pharmaceutical services? | Why? | I3 relative advantage III 8c relative priority |
| 3 | In your opinion, what will be the role of community pharmacists in 10 years’ time? | More as health professionals, focusing on pharmaceutical services? Or a focus on logistics and dispensing? | IV 8 values & motivation |
|  | Would you like to add anything? | You’re very welcome to contact me at any time. | Would you like to add anything? |
|  | Many thanks for your time! You have been most helpful. |  | Thanks & Goodbye |
